# Membranous nephropathy associated with profound hypothyroidism

**DOI:** 10.1002/ccr3.2573

**Published:** 2019-12-04

**Authors:** Girish Singhania, Namrata Singhania

**Affiliations:** ^1^ Department of Hospital Medicine CHI St. Vincent Infirmary Little Rock AR USA; ^2^ Department of Hospital Medicine Mount Carmel East Hospital Columbus OH USA

**Keywords:** acute renal failure, angiotensin converting enzyme inhibitors, hypothyroidism, proteinuria

## Abstract

Membranous nephropathy can be primary or secondary. Although common causes of secondary MN are malignancy and lupus, hypothyroidism was rarely reported. Providers should check thyroid function tests in patients with unexplained nephrotic proteinuria.

## Graphical abstract

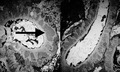

## 1 INTRODUCTION

We report a case of membranous nephropathy (MN) in a patient with hypothyroidism who presented with acute renal failure and nephrotic range proteinuria. Our case raises awareness regarding severe kidney disease in the setting of profound hypothyroidism and checking thyroid function test in patients with unexplained kidney injury and proteinuria.

Hypothyroidism is one of the common endocrine disorders in the world. Disorders of thyroid function have been rarely linked to alteration in kidney function as well as development of immune‐mediated glomerular injury. Membranous nephropathy, for example, has been associated with both autoimmune thyroiditis (Hashimoto's disease) 1 and Graves' disease.^2^ The mechanism linking thyroid disease and kidney injury is not well established. Here, we report a case of secondary MN associated with untreated profound hypothyroidism.

## 2 CASE PRESENTATION

### 2.1 History and examination

A 64‐year‐old Hispanic man with no known history of kidney problems presented with dyspnea, fatigue, and lower extremity (LE) edema. There was no positive history of loss of appetite or significant weight loss. His family history was negative for any kidney disease or any autoimmune disease. He was nonsmoker and drinks socially. He does not have any history of illicit drug use. He was not taking any nonsteroidal anti‐inflammatory drugs, proton pump inhibitors, antibiotics, or any other known nephrotoxins at home. Examination showed blood pressure of 166/87 mm Hg and edema of scrotum and bilateral LE. His lungs were clear to auscultation bilaterally, and his pulse has regular rate and rhythm.

### 2.2 Initial work‐up

Laboratory indices showed normal sodium and potassium levels, serum creatinine was 3.1 mg/dL (normal baseline), proteinuria 4.5 g per gram of creatinine, albumin 2 g/dL, and hemoglobin 8.9 g/dL with normal iron studies. Thyroid‐stimulating hormone was 99.28 mIU/L (normal range 0.27‐4.20 mIU/L), free thyroxine (T4) 0.52 ng/dL (normal range 0.93‐1.70 ng/dL), and total triiodothyronine (T3) 45 ng/dL (normal range 80‐200 ng/dL). His urine microscopy was negative for dysmorphic red blood cells.

### 2.3 Differential diagnosis and further work‐up

At this point, with his history of edema and laboratories suggesting acute kidney injury, hypoalbuminemia, and nephrotic range proteinuria, we were inclined toward the glomerular cause of his kidney injury. We had focal segmental glomerulonephritis, membranous nephropathy, and rapidly progressive glomerulonephritis associated with antineutrophil cytoplasmic antibodies (ANCA) or antinuclear antibodies (ANA) as seen in lupus or hepatitis C‐related kidney disease in our mind. Further work‐up was ordered to narrow down the differential diagnosis. Complement levels C3 and C4 were normal. Antinuclear, antineutrophil cytoplasmic, antiglomerular basement membrane, thyroglobulin, and thyroid peroxidase antibodies were negative. Protein electrophoresis was negative. Viral serology (HIV and hepatitis) was negative, and hemoglobin A1c was 5.1%. Chest radiograph was negative for any acute cardiopulmonary pathology. Ultrasound showed normal kidneys, and transthoracic echocardiogram revealed ejection fraction of 20%‐25%.

### 2.4 Pathology

**Figure 1:**
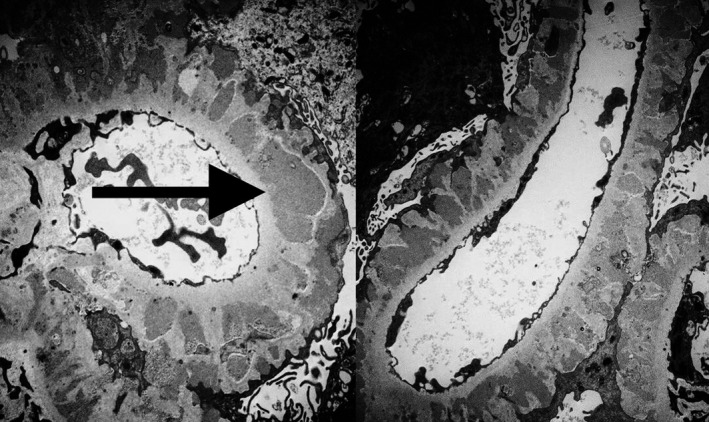
Electron microscopy of kidney tissue showing membranous nephropathy with thickening of glomerular basement membrane due to presence of intramembranous deposits (arrow)

A kidney biopsy was performed, and electron microscopy showed membranous nephropathy stage II‐III with features favoring secondary forms like mesangial proliferation. There were subepithelial and intramembranous deposits with thickening of glomerular basement membrane (Figure 1).

### 2.5 Treatment

The patient was diagnosed with secondary membranous nephropathy likely related to profound hypothyroidism. He was treated with intravenous thyroid hormone replacement with rapid improvement in his symptoms. He was also started on angiotensin converting enzyme inhibitors (ACEI) and diuretics which helped with proteinuria and volume overload, respectively.

### 2.6 Outcome and follow‐up

In the follow‐up clinic visit, his swelling was significantly improved, and his creatinine was close to baseline at 1.1 mg/dL. His proteinuria was also down to non‐nephrotic range (0.46 g/g of creatinine) from nephrotic range previously. His serum albumin also improved to 3.6 g/dL. The patient was also referred to an endocrinologist for management of his hypothyroidism.

## 3 DISCUSSION

MN is among the most common causes of the nephrotic syndrome in nondiabetic adults, accounting for up to one‐third of biopsy diagnoses in some regions. This can be divided into two groups, primary or secondary. Primary MN occurs by itself, and majority of cases are associated with autoantibody against phospholipase A2 receptor. Secondary MN occurs due to other diseases like autoimmune like lupus, infections, malignancy, or hypothyroidism (rare) like in our case.

Ehrenreich et al^3^ have described four ultrastructural stages of MN: Stage I is characterized by the presence of scattered or more regularly distributed small immune complex‐type electron‐dense deposits in the subepithelial zone. Stage II is characterized by projections of basement membrane material around the subepithelial deposits. Stage III is characterized by new basement membrane material surrounds the deposits. Stage IV is characterized by the loss of electron density of the deposits, resulting in irregular electron‐lucent zones within an irregularly thickened basement membrane. Biopsy findings of secondary MN are characterized by mesangial and/or subendothelial deposits which are rarely seen in primary MN. Secondary membranous due to lupus nephritis can have positive immunofluorescence for immunoglobin (Ig)A, IgM, IgG, complement C3, and C1q “full house pattern.”

Thyroid disease has previously been reported to have a link with development of glomerulonephritis.^4^ Li et al reviewed 317 patients with nephrotic syndrome and found that the most common renal pathology in patients with hypothyroidism was membranous nephropathy followed by minimal change disease.^5^ While autoimmune pathways have been proposed as potential mechanisms underlying this association, so far, no precise antibody has been identified.^2^, ^6^ Interestingly, we did not find any autoimmune cause for profound hypothyroidism in our patient while he presented with secondary MN. Treatment in patients with secondary MN is mainly focused on cessation of the offending agent or effective treatment of the underlying disease. Sometimes, immunosuppressive agents are required for treatment depending on the pathophysiology of MN, for example, in lupus‐related MN. Control of blood pressure and proteinuria is crucial and is achieved by use of ACEI. Diuretics are required to control volume overload. Our case raises awareness regarding severe kidney disease in the setting of profound hypothyroidism and checking thyroid function test in patients with unexplained kidney injury and proteinuria. Early treatment with intravenous and later, oral thyroid hormone replacement is important for successful renal recovery.

## CONFLICT OF INTEREST

Authors do not have any conflict of interest to disclose.

## AUTHOR CONTRIBUTIONS

GS: saw the patient, prepared the manuscript, and did literature review. NS: reviewed the manuscript and literature.
